# Acute High Level Noise Exposure Can Cause Physiological Dysfunction in Macaque Monkeys: Insight on the Medical Protection for Special Working Environmental Personnel

**DOI:** 10.3390/healthcare9070840

**Published:** 2021-07-02

**Authors:** Weijia Zhi, Haoyu Wang, Yong Zou, Xinping Xu, Ning Yu, Yuyang Zhu, Yanling Ren, Lizhen Ma, Yefeng Qiu, Xiangjun Hu, Lifeng Wang

**Affiliations:** 1 Beijing Institute of Radiation Medicine, 27 Taiping Road, Beijing 100850, China; zhi.weijia@163.com (W.Z.); smart106@126.com (H.W.); tjuzouyong@163.com (Y.Z.); xuxp2012@sina.com (X.X.); gfszhuyuyang@yeah.net (Y.Z.); malizhen0906487@sina.com (L.M.); 2 Chinese PLA General Hospital, Chinese PLA Medical School, College of Otolaryngology Head and Neck Surgery, Beijing 100853, China; yuning@uky.edu; 3 Laboratory Animal Center of the Academy of Military Medical Sciences, 20, Dongda Street, Beijing 100071, China; 1989-ryl-1989@163.com

**Keywords:** acute high level noise exposure, macaque monkeys, auditory brainstem response, auditory P300, electrocardiogram

## Abstract

The high level noise caused by intense acoustic weapons and blasting is a common source of acute acoustic trauma faced by some special environmental personnel. Studies have shown that high level noise can cause auditory and non-auditory effects. However, there are few reports on the biological effects, especially the non-auditory effects of acute high level noise exposure in simulated special working environments, and the great differences between experimental animals and human beings make it difficult to extrapolate from research conclusions. In this study, macaque monkeys were used to detect the effects of acute high level noise exposure on hearing, cognition, and cardiovascular function. Auditory brainstem response, auditory P300, and electrocardiogram (ECG) of macaque monkeys were measured. Results showed that acute high level noise exposure caused permanent hearing threshold shifts; partial hearing loss which couldn’t recover to normal levels in the detection period; pathological changes in T wave and QRS complexes; and large fluctuations in cognitive ability after exposure, which finally recovered to normal. These alterations may be a combination of effects caused by stress-induced neuroendocrine dysfunction and mechanical damage of auditory organs. To elaborate the exact mechanism, further studies are still needed. Meanwhile, positive measures should be taken to reduce the incidence of acute high level noise injury.

## 1. Introduction

Sound is a mechanical wave produced by physical vibrations. It can be classified as infrasound (<20 Hz), audible (20 Hz to 20 kHz), and ultrasonic (>20 kHz) sound; audible sound can also be classified as low frequency (<400 Hz), medium frequency (400~1000 Hz), and high frequency (>1000 Hz) [1]. In terms of intensity, it can be classified into high intensity and low intensity. A sound pressure level (SPL) over 120 Decibels (dB) is a high intensity sound. The ear is an extremely sensitive target organ to unexpected sound, especially high intensity sound. Usually the higher the intensity, the greater the damage is. Even in the case of the same intensity, frequency differences also have different effects on hearing; usually high frequency is more harmful than low frequency. The high level noise caused by intense acoustic weapons and blasting is a common source of acute acoustic trauma faced by relevant environmental personnel [2,3]. Since the 1990s, the intense acoustic weapon has become a hot research field for the world’s military powers, led by the United States, and has been applied in troops. As a typical non-lethal weapon, the intense acoustic weapon has been widely used in the fields of land and sea defense, anti-terrorism, stability maintenance, and riot control, etc. It has achieved remarkable combat effects in the Iraq War and Afghanistan War. In the past 20 years, the intense acoustic weapon has been developed towards multi-functionality, intelligence (with powerful physiological and psychological control), miniaturization, long-distance, multiplatform compatibility, and good environmental adaptability, showing a great potential for application. At the same time, the acute high level noise exposure caused by blasting during military actions or some construction engineering also poses a health threat to related personnel [4].

Studies have shown that high level noise can cause auditory effects, such as hearing damage, including damage on hair cells and neural afferent pathways; secondary extra auditory effects; and non-auditory effects on the nervous system, endocrine system, and cardiovascular system [5,6]. When exposed to high level noise of 90~120 dB (A) at a low frequency (5~200 Hz) for 1 min, it will produce extreme irritability and an upset feeling; infrasound of 110~130 dB (A) will cause intestinal pain and nausea; when the SPL reaches 140~150 dB (A), severe trauma on the body and tissue will occur. A sound of SPL > 170 dB (A) will cause damage which is similar to that made by an instantaneous shock wave [7,8,9]. In military actions such as anti-terrorism and anti-riot actions, military personnel on the frontline are vulnerable to injury from intense acoustic weapons. In addition, military personnel and some special workers are often exposed to high level noise in a blasting environment. If not protected properly, the health of relevant personnel is bound to be affected, for example, temporary or permanent threshold shifts, hearing loss, and secondary extra auditory effects [2,10]. The ability to hear and recognize combat-relevant sounds is a vital component to situational understanding and provides a tactical advantage [2]. Acute acoustic trauma could impair situational awareness (including communication, positioning, and threat recognition) of personnel such as military and construction workers [11], and adversely affect combat and working readiness [12]. Therefore, in order to improve combat ability and the working achievements, it is indispensable to explore the biological effects of acute high level noise exposure. However, the previous study on the biological effects of high level noise exposure mainly studied small animals such as pigs and rabbits. While the human ear is most sensitive to sounds of 2~4 kHz, the auditory system of monkey is most similar to humans, so in this research, macaque monkeys are adopted to figure out the effects of high level noise exposure on the auditory, cognitive, and cardiovascular system, in order to provide a powerful basis of extrapolation about acute high level noise exposure effects, as well as lay a foundation for safe application of relevant equipment, for example, intense acoustic weapons, and appropriate protection for the auditory and non-auditory health of workers exposed to acute high level noise in special working environments.

## 2. Materials and Methods

### 2.1. Experimental Animals and Groups

Three healthy male macaque monkeys (numbered M1, M2, and M3) aged from 4~5 years old, weighing about 4 kg, were used in the experiment, and were provided by Experimental Animal Center of Beijing Institute of Radiation Medicine. All protocols were approved by the Institutional Animal Care and Use Committee of the Beijing Institute of Radiation Medicine. All the methods in this study were performed in accordance with the relevant guidelines and regulations. The ethics review number was IACUC-DWZX-2019-515. The genetic and immunological background of these monkeys is clear, common infectious diseases tests were negative, and there was no history of ear disease or exposure to toxic drugs. Each monkey was kept in a single cage, the temperature was 18~28 °C and the humidity was 40~70%. Mechanical ventilation was 8~10 times/h; Illuminance was 150~300 Lux; and the lighting time was 12 h/12 h.

### 2.2. High Level Noise Exposure

Monkeys were fixed on monkey chairs and exposed to high level noise of 160 dB, 1~4 kHz mixing frequency which the sound was mixed by Cool Edit software at different frequency points ranging from 1 to 4 kHz for 10 min. The main purpose of this processing is to simulate the high level noise in a special environment such as a battlefield or construction environment. Before and 0 h, 7 d, 14 d, 28 d, and 90 d after exposure, auditory brainstem evoked potential was detected to evaluate hearing impairment. P300 and electrocardiogram (ECG) data were collected before and 0 h, 7 d, 14 d, 28 d, and 180 d after exposure to evaluate the effects of high level noise on the physiological function of macaque monkeys. The difference of detection time was mainly because of the difficulty of experimental coordination.

### 2.3. Measurement of ECG Indicators

Monkeys were fixed on monkey chairs awake. The needle electrodes were used and put in the right upper limb and bilateral lower limbs subcutaneously, the ECG amplifier (MP-150, Biopac Systems, Goleta, CA, USA) was connected, then the parameters of standard II lead electrocardiogram (ECG) including heart rate (HR); wave amplitude value of P, R and T wave; P—R interphase; T wave interphase; Q—T and QRS interphase were recorded.

### 2.4. Measurement of Auditory Brainstem Response

The macaque monkeys were anesthetize by ketamine (10 mg/kg), and auditory brainstem response (ABR) hearing threshold was detected by Intelegent Hearing Systems (Smart EP, Miami, FL, USA). The recording electrode was inserted into the scalp to the bone surface at the midpoint between the leading edge of the ears, the reference electrode was inserted subcutaneously into the earlobe of the test ear, and the grounding electrode was inserted into the tip of the nose. “Click” was used for monaural stimulation, the duration was 100 ms, the stimulation frequency was 19.3/s, and the collected signal was superposed 1024 times. The sound was fed by built-in earphones, and the low-pass filter was 50 Hz, the high-pass filter was 3000 Hz. The stimulus intensity started from 100 dB, and decreased 10 dB every step until the wave V disappears, and the ABR threshold of wave V was recorded.

### 2.5. Collection and Analysis of Auditory P300 Event-Related Potential

Experiments were conducted in the acoustic and electrical shielding room, and the macaque monkey was fixed on the monkey chair awake, the needle electrode was inserted into the scalp at the corresponding position, including PZ, CZ, and FZ. The reference electrode was between CZ and PZ, the position of electrooculogram (EOG) acquisition is at the orbit, the grounding electrode was behind the left ear. E-prime 2.0 was used for programming. Auditory stimuli were given by E-prime 2.0 software in a simple Oddball paradigm, sound intensity was 70 dB. EEG data were collected and recorded by Curry 8 and SynAmps2, and then analyzed offline.

Stimulation parameter settings: the probability ratio of target stimulation sound and non-target stimulation sound was 40:160, frequency of the stimulation sounds were 2000 Hz and 1000 Hz, both were short pure tones, repetition time was 1 Hz. Rising and falling edge of non-target stimulation were both 102.5 ms, platform period was 779.8 ms. Those for target stimulation were 101.1 ms and 797.5 ms respectively.

### 2.6. Statistical Analysis

Means ± standard deviation (SD) are adopted for statistical analysis. Comparisons between groups before and after exposure were analyzed by ANOVA. Pairwise comparison among groups was done with q-test. The accepted level of significance for all tests was *p* < 0.05.

## 3. Results

### 3.1. Effects of High Level Noise on Auditory Brainstem Response in Macaque Monkeys

The scalp-recorded activation of the nerve fibers in the auditory nerve and brainstem is called the auditory brainstem response (ABR), sometimes called brainstem auditory evoked potentials (BAEPs) [13]. ABR can be used to estimate the auditory ability and can determine the type and extent of hearing loss. It is an electrical response induced by a short sound, which reflects the hearing threshold in the high frequency range of 2~4 kHz. Wave V disappears last among the waves of ABR, which allows the collection of objective data without the subject’s response because it is not affected by subjects’ subjective consciousness, sleep, or drugs. The threshold of wave V, which is usually the minimum intensity of a short sound inducing wave V, has been widely used as an objective indicator of auditory ability [14].

**Figure 1:**
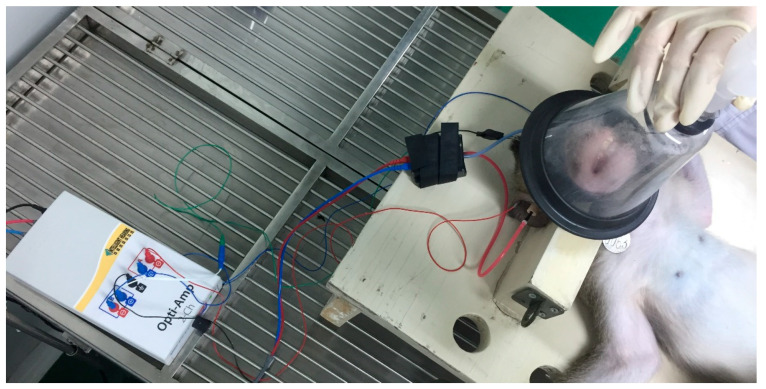
Experimental photos of ABR detection.

**Figure 2:**
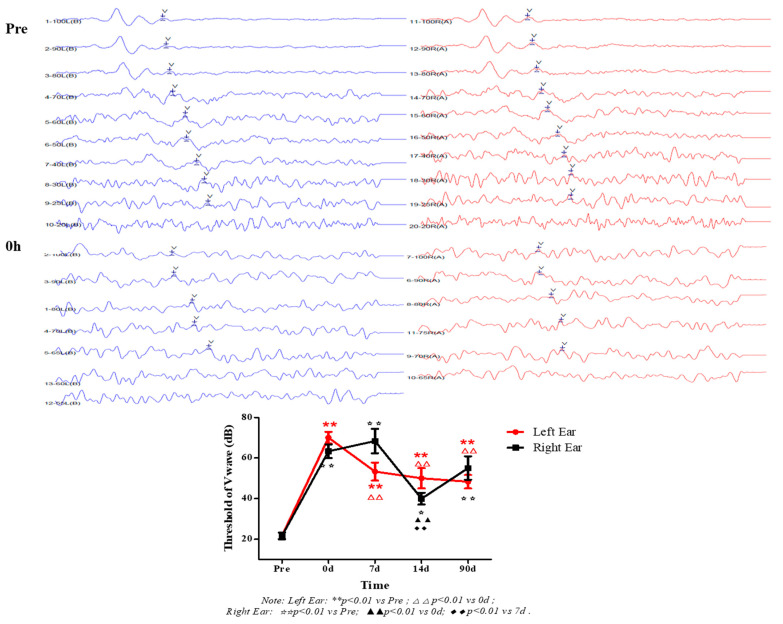
Effects of high level noise exposure on auditory brainstem response in macaque monkeys. Pre and 0 h: ABR waves before and immediately after high level noise exposure, the position indicated by the arrow is wave V of ABR; the line chart is the threshold of wave V at each detection time point.

Figure 1 showed that the ABR signal of anesthetized macaque monkeys was being collected. In Figure 2, before exposure to high level noise, the average threshold values of wave V (referred to as “threshold” hereinafter) for both ears were 21.67 dB, immediately after exposure, the threshold lifted for at least 40 dB (*p* < 0.01 vs. Pre). For the left ear, thresholds of 7 d, 14 d and 28 d after exposure decreased compared with 0 h after exposure (*p* < 0.01), but still higher than that before exposure (*p* < 0.01). For the right ear, the threshold of each detecting time point after exposure was significantly higher than that before exposure (*p* < 0.01 or *p* < 0.05). At 14 d after exposure, the threshold was lower than 0 h and 7 d after exposure (*p* < 0.01), but increased again at 90 d after exposure to a certain extent but showed no significance compared to 0 h, 7 d and 14 d.

### 3.2. Effects of High Level Noise on ECG of Macaque Monkeys

The electrocardiogram (ECG) is one of the important indicators in studies about noise-related cardiovascular diseases.

**Figure 3:**
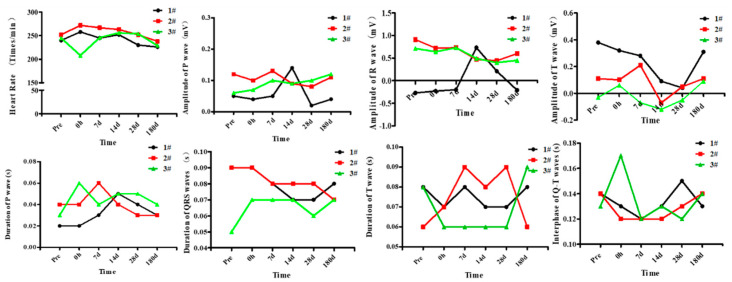
Effects of high level noise on ECG of macaque monkeys.

Before exposure, all the three monkeys showed sinus rhythm, uniform heart rate, and spike shaped upright P wave. The QRS waves of monkey M1 showed a drastic change: before, 0 h, and 7 d after exposure it was QR type; while at 14 d after exposure, it was QRS type; at 28 d it was RS type; and at 180 d it was back to QR type again. The T wave was upright at each time point. For monkey M2, respiratory arrhythmia occurred at 7 d after exposure. The QRS waves were QRS type, and the T wave was low and flat at 14 d after exposure, and was upright at other detection time points. The QRS waves of monkey M3 were QRS type, and the T wave was flat or inverted after exposure. Figure 3 showed the fluctuation of the ECG parameters of three monkeys at each detection time point. Table 1 showed the value (M ± SD) of the ECG parameters at each time point. The data showed great individual variation, M1 showed great difference in amplitude value of P and R waves. For QRS, T and QT interphase, M3 was different with other monkeys.

### 3.3. Effects of High Level Noise on Auditory Event-Related Potential (P300) in Macaque Monkeys

**Figure 4:**
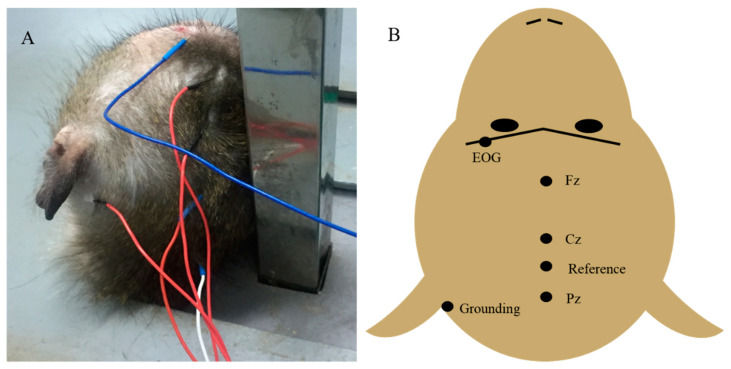
Experimental photos of ABR detection. (**A**). Photos of macaque monkeys during P300 recording, (**B**). Individual electrodes were inserted into the scalp according to the illustration.

P300 is a family of positive components in the frontal and parieto-occipital lobes appearing in about 300 milliseconds. It reflects the attention and discrimination ability of task-related targets and cues during stimulus processing, and has been widely used in the study of neurological and psychiatric cognitive dysfunction [15,16]. Figure 4 showed the photos of macaque monkeys during P300 recording, and the diagram of how the individual electrodes were placed.

**Figure 5:**
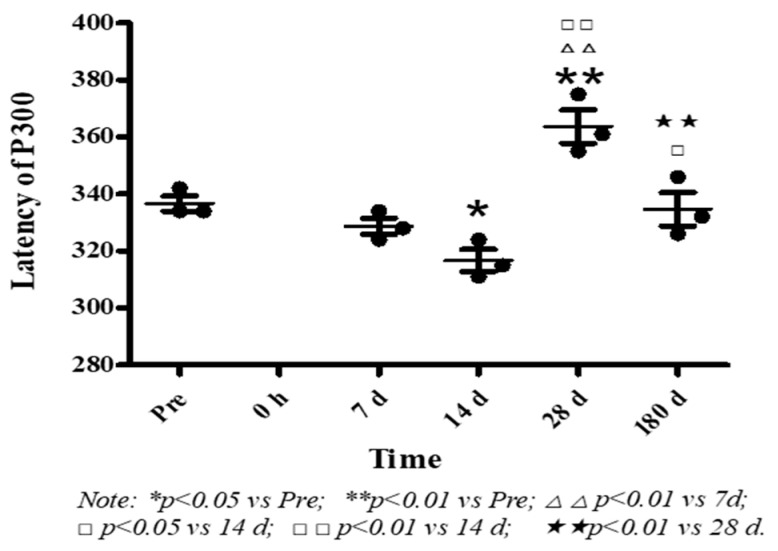
Effects of high level noise on auditory p300 of macaque monkeys.

In Figure 5, the average auditory P300 latency of macaque monkey was 336.67 ± 4.6 ms before exposure. Immediately after exposed to high level noise, there was no P300 component detected in corresponding time windows, and it appeared again 7 d after exposure. At 14 d after exposure, the latency was significantly shorter than that before exposure (*p* < 0.05), went up at 28 d after exposure (*p* < 0.01), and decreased at 180 d to a level between 14 d and 28 d (*p* < 0.01 or *p* < 0.05).

## 4. Discussion

The ear is the most vulnerable target for intense acoustic trauma [17]. In some special working environments, such as military and blasting, related engineering, personnel are often exposed to a variety of potentially harmful noise [18]. The U.S. Department of Defense sets 140 dB as the loudest pressure level for impulse noise acceptable to the ears without protected operators [19]. High level noise ≥ 140 dB can cause acute acoustic trauma (AAT), the high-power waves can cause auditory system injury [10], including injuries in sensitive structures of the middle and inner ears, such as the eardrum, ossicle chain, cochlea, and vestibular system, even further resulting in perforation of the eardrum, separation of the ossicle, conduction, sensorineural or mixed hearing loss, vertigo, and tinnitus [20,21]. This has become a common public health problem among related personnel, which seriously impairs work and combat effectiveness. At the same time, the environments the above personnel worked in are extremely dangerous, and usually involve high level noise exposure. However, it is difficult to measure the sound in the wild accurately, and many factors restricted simulation in a laboratory, thus the research about high level noise exposure in special working environments was much less than that in industry [22]. The high level noise caused by intense acoustic weapons and acute blasting could threaten the physical and mental health of operators and accidentally exposed personnel [23]. Previous studies on the biological effects of acute high level noise exposure mostly focused on the auditory damage, while the non-auditory effects were less studied. Studies have shown that stress response, cognitive and cardiovascular dysfunction are typical non-auditory effects of long-term exposure to occupational noise [5,24,25,26]. Do these effects exist in acute high level noise exposure? With this question, focusing on auditory and non-auditory effects, macaque monkeys were studied.

In threshold-seeking auditory brainstem response (ABR) evoked response audiometry (ERA) tests the response is occasionally seen to be superimposed on an upwards or downwards sloping baseline [27]. Ideally, the ECG between the active electrode and the reference electrode of ABR is consistent. However, the ECG at each electrode of ABR couldn’t be completely eliminated due to the neck structure of the subject. In this research, the threshold of wave V was adopted to evaluate the influence of high level noise on macaque monkeys’ auditory ability; the results showed it was significantly elevated immediately after exposure, suggesting partial hearing loss occurred. Although the thresholds at the following testing time points fluctuated, it didn’t recover to the normal level. Thus, the high level noise caused permanent partial hearing loss or a permanent shift of the auditory threshold for 26.7 dB in the macaque monkeys. Studies have shown that mechanical impairment dominates in permanent hearing loss, and reversible metabolic changes may explain the temporary threshold shifts [28]. In this research, the short-term decrease in hearing threshold in macaque monkeys after exposure may be related to the reversible metabolic changes in the inner ear, while their permanent partial hearing loss may be attributed to mechanical injuries such as cochlear hair cell loss and peripheral neuron degeneration.

It is reported acoustics can pose impacts on the gastrointestinal tract [29], respiratory system [30], immune system [31], reproductive system [32], nervous system [33], and other systems. Among them, the influence on the cardiovascular system is particularly prominent. Long-term exposure to sound of more than 90 dB (A) can lead to severe damage to the auditory system, resulting in neurasthenia, headache, hypertension, and other diseases. Noise exceeding the pain threshold (100 dB (A)) will cause ear swelling and pain. Noise above 115 dB (A) could impair the function of the cerebral cortex. Cardiac resonance may occur when noise is above 175 dB(A) and may lead to death [5]. The effects of noise on the cardiovascular system may be long-term and sustainable. As a possible risk factor of cardiovascular disease, noise can accelerate cardiac senescence and increase the incidence of myocardial infarction. However, compared with the studies on the effects of noise on the auditory system, noise damage to the non-auditory systems, especially the nervous and cardiovascular system, has not drawn enough attention.

In this study, focusing on changes in cardiovascular system and neurocognitive ability, electrocardiogram and auditory P300 event-related potential were detected to explore the effects of acute high level noise exposure on non-auditory system.

It is reported that the ECG changes before hearing loss and the abnormalities are dose-dependent with cumulative noise exposure, thus they can be used as an indicator of injury. The incidence of ECG abnormalities among workers exposed to occupational noise were 2.27 times higher than those of a control group, and the correlation between the incidence and exposure level was statistically significant [5]. However, the effects of acute high level noise exposure on the cardiovascular system are rarely reported. In this study, after acute exposure to high level noise, the ECGs of the monkeys showed various abnormal changes, mainly manifesting as abnormal QRS complexes and T wave, and occasionally abnormal heart rate. Research has shown that noise as a stressor could generally induce a range of responses, especially in cardiovascular systems. Animal and human studies have also confirmed that noise is a risk factor for cardiovascular disease which can cause fluctuations in blood pressure, and intense occupational noise exposure can accelerate or reduce heart rate. This might be related to the autonomic nerve chaos caused by the stress-related hormone, and organic injury in the heart may also be involved. For the further conformation, morphological studies and expanded samples are still needed. P300 is an endogenous component that is independent from physical properties of the stimulus [34]. Amplitude of P300 is directly related to memory, and may be more sensitive to the amount of attention resources occupied in the task [35]. In other words, the amplitude may be related to the frequency of target stimulus, but its significance is still controversial. The latency of P300 reflects the rate of stimulus classification [36], and is generally independent of explicit response and behavioral response [37,38]. Neuropsychological tests of attention and immediate memory have shown that the shorter the P300 latency, the better the cognitive performance [39]. In this study, due to poor compliance of the subjects, the macaque monkeys were only stimulated with Oddball paradigm in the test process, and were not required to respond to the target stimulus. A sound of 70 dB was used as the stimulus because this sound pressure level is easily perceived by most human subjects and represents a common level of human language [16]. According to the statistics of the World Health Organization, the loss of healthy life years caused by noise is mainly related to annoyance, cognitive impairment, and sleep disorders, while mental illnesses such as post-traumatic stress disorder after acute exposure to high level noise are also potential sequelae [40,41]. According to the results of this study, the P300 component of macaque monkeys could be successfully induced before exposed to high level noise. However, it was not detected immediately after exposure, which may be a result of the dramatic rise of the hearing threshold. At 7 d after exposure, alongside the hearing threshold recovering to a certain extent, P300 appeared again, and the latency shortened temporarily at 14 d after exposure, suggesting cognitive ability was enhanced which was possibly due to stress. At the other testing time points, the cognitive ability of macaque monkeys significantly decreased, manifested as lengthening of the latency. The cognitive ability returned to the pre-exposure level at 180 d after exposure. The above cognitive fluctuations may be caused by neuroendocrine system dysfunction due to high level noise exposure, and further experiments are still needed for the exact mechanism.

## 5. Limitations

In this study, macaque monkeys, which are closer to human species than other small animals, were selected as the research objects, which laid a foundation for extrapolating the physiological effects of acute high level noise exposure. However, there are still some limitations. For example, in order to ensure the homogeneity of the tested animals, the number of monkeys meeting the restrictions of this study (4~5 years old, weighing about 4 kg) is limited, only 3 monkeys could be included in this experiment. We observed the auditory and non-auditory effects on macaque monkeys after acute high level noise exposure. However, for the sake of animal welfare protection and ethical justification, we did not explore the specific morphological changes and pathological mechanisms of the above manifestations. These are the shortcomings to be improved.

## 6. Conclusions

In conclusion, high level noise of 160 dB for 10 min can cause partial hearing loss, cardiovascular and neurocognitive dysfunction in exposed monkeys. It is reported that the potential mechanism of noise-induced mental stress mainly focuses on the stress response of the sympathetic nerve and endocrine (such as increased stress hormone levels, blood pressure and heart rate, etc.), which in turn impair cerebrovascular function, promoting the development of cerebrovascular diseases such as stroke, arterial hypertension, ischemic heart disease, and myocardial infarction. The cardiovascular and mental disease induced by long-term exposure to low-dose noise has cumulative effects, however, the cognitive and cardiovascular dysfunction caused by acute exposure to high level noise is more likely to be a combined effect of sustainable stress responses and mechanical damage in auditory organs: psychological abnormalities induced by stress may aggravate subjective negative feelings of auditory dysfunction, and then feedback at the physiological level, while partial hearing loss continues the negative feedback, eventually seriously affecting the health status and quality of life of the exposed persons.

As a common trauma in special environments, such as military actions and blasting, acute high level noise should be protected correctly and diagnosed as soon as possible to reduce the incidence. Hearing Protection Devices (HPDs) have been proved to be an effective means to prevent and reduce sequelae of acute high level noise injury and other complications of non-auditory effects. Effective measures should be taken, such as equipping potential exposed personnel with protective devices and training them in their use, as well as having their auditory ability checked, and regularly conducting noise protection consultations. Only in this way can working effectiveness be improved and the physical and mental health of the exposed personnel be ensured.

## Figures and Tables

**Table 1:** Value of the ECG parameters at each time points (M ± SD).

Detection Time	Hear Rate (bpm)	P (mV)	R (mV)	T (mV)	P (s)	QRS (s)	T (s)	Q-T (s)
**Pre**	245.67 + 6.03	0.08 ± 0.04	0.45 ± 0.63	0.15 ± 0.21	0.03 ± 0.01	0.08 ± 0.02	0.07 ± 0.01	0.14 ± 0.01
**0 d**	246.00 + 33.65	0.07 ± 0.03	0.38 ± 0.53	0.16 ± 0.14	0.04 ± 0.02	0.08 ± 0.01	0.07 ± 0.01	0.14 ± 0.03
**7 d**	252.67 + 12.42	0.09 ± 0.04	0.42 ± 0.54	0.14 ± 0.19	0.04 ± 0.02	0.08 ± 0.01	0.08 ± 0.02	0.12 ± 0
**14 d**	257.00 + 5.57	0.11 ± 0.03	0.56 ± 0.14	(−)0.03 ± 0.11	0.05 ± 0.01	0.07 ± 0.01	0.07 ± 0.01	0.13 ± 0.01
**28 d**	245.33 + 13.32	0.07 ± 0.04	0.35 ± 0.12	0.01 ± 0.06	0.04 ± 0.01	0.07 ± 0.01	0.07 ± 0.02	0.13 ± 0.02
**180 d**	231.00 + 6.24	0.09 ± 0.04	0.28 ± 0.43	0.17 ± 0.12	0.03 ± 0.01	0.07 ± 0.01	0.08 ± 0.02	0.14 ± 0.01

## Data Availability

The data presented in this study are available on request from the corresponding author. The data are not publicly available due to it will also be used in subsequent studies.

## References

[1] Walstead S., Deane G.B. (2016). Intensity statistics of very high frequency sound scattered from wind-driven waves. J. Acoust. Soc. Am..

[2] Yehudai N., Fink N., Shpriz M., Marom T. (2017). Acute Acoustic Trauma among Soldiers during an Intense Combat. J. Am. Acad. Audiol..

[3] Hemel N.V., Verzijlbergen J.F. (2016). Acoustic shock waves, a new weapon against angina?. Neth. Heart J..

[4] Lie A., Skogstad M., Johannessen H.K.A., Tynes T., Mehlum I.S., Nordby K.C., Engdahl B., Tambs K. (2016). Occupational noise exposure and hearing: A systematic review. Int. Arch. Occup. Environ. Health.

[5] Yang Y., Zhang E., Zhang J., Chen S., Yu G., Liu X., Peng C., Lavin M.F., Du Z., Shao H. (2018). Relationship between occupational noise exposure and the risk factors of cardiovascular disease in China. Medicine.

[6] Dijk F. (1990). Epidemiological research on non-auditory effects of occupational noise exposure. Environ. Int..

[7] Zajamšek B., Hansen K.L., Doolan C.J., Hansen C.H. (2016). Characterisation of wind farm infrasound and low-frequency noise. J. Sound Vib..

[8] Oerlemans S. (2015). Effect of wind shear on amplitude modulation of wind turbine noise. Int. J. Aeroacoustics.

[9] Baliatsas C., van Kamp I., van Poll R., Yzermans J. (2016). Health effects from low-frequency noise and infrasound in the general population: Is it time to listen? A systematic review of observational studies. Sci. Total Environ..

[10] Lee J., Bowley D.M., Miles J., Muzaffar J., Orr L. (2020). The Downrange Acoustic Toolbox: An Active Solution for Combat-Related Acute Acoustic Trauma. J. Spec. Oper. Med. Peer Rev. J. Sof. Med. Prof..

[11] Milford C., Theobald M.R., Nemitz E., Hargreaves K.J., Horvath L., Raso J., Dämmgen U., Neftel A., Jones S.K., Hensen A. (2009). Ammonia fluxes in relation to cutting and fertilization of an intensively managed grassland derived from an inter-comparison of gradient measurements. Biogeosci. Discuss..

[12] Medina-Garin D.R., Dia A., Bedubourg G., Deparis X., Berger F., Michel R. (2016). Acute acoustic trauma in the French armed forces during 2007–2014. Noise Health.

[13] Eggermont J.J. (2019). Auditory brainstem response. Handb Clin Neurol.

[14] Holme R., Steel K. (2004). Progressive Hearing Loss and Increased Susceptibility to Noise-Induced Hearing Loss in Mice Carrying a Cdh23 but not a Myo7a Mutation. J. Assoc. Res. Otolaryngol. Jaro.

[15] Polich J. (2007). Updating P300: An integrative theory of P3a and P3b. Clin. Neurophysiol..

[16] Polich J. (2004). Clinical application of the P300 event-related brain potential. Phys. Med. Rehabil. Clin. N. Am..

[17] Darley D.S., Kellman R.M. (2010). Otologic considerations of blast injury. Disaster Med. Public Health Prep..

[18] Hammill T., Le Prell C., Allen F.R., Kujawa S., Kil J. (2016). Temporary and Permanent Noise-induced Threshold Shifts: A Review of Basic and Clinical Observations. Otol. Neurotol. Off. Publ. Am. Otol. Soc. Am. Neurotol. Soc. Eur. Acad. Otol. Neurotol..

[19] Occupational Safety and Health Administration (1983). Guidelines for Noise Enforcement: Appendix A (OSHA Directive CPL 2–2.35 A).

[20] Shah A., Ayala M., Capra G., Fox D., Hoffer M. (2014). Otologic assessment of blast and nonblast injury in returning Middle East-deployed service members. Laryngoscope.

[21] Remenschneider A.K., Lookabaugh S., Aliphas A., Brodsky J.R., Devaiah A.K., Dagher W., Grundfast K.M., Heman-Ackah S.E., Rubin S., Sillman J. (2014). Otologic outcomes after blast injury: The Boston Marathon experience. Otol. Neurotol..

[22] Smalt C.J., Lacirignola J., Davis S.K., Calamia P.T., Collins P.P. (2016). Noise dosimetry for tactical environments. Hear. Res..

[23] Zhang J. (2019). Blast-induced tinnitus: Animal models. J. Acoust. Soc. Am..

[24] Sheppard A., Ralli M., Gilardi A., Salvi R. (2020). Occupational Noise: Auditory and Non-Auditory Consequences. Int. J. Environ. Res. Public Health.

[25] Muenzel T., Schmidt F.P., Sebastian S., Herzog J., Daiber A., Sørensen M. (2018). Environmental Noise and the Cardiovascular System. J. Am. Coll. Cardiol..

[26] Choi S.H., Choi C.H. (2015). Noise-Induced Neural Degeneration and Therapeutic Effect of Antioxidant Drugs. J. Audiol. Otol..

[27] Lightfoot G. (2017). Sloping ABR baselines and the ECG myogenic artefact. Int. J. Audiol..

[28] Kellerhals B. (1987). Acute acoustic trauma. Acta Oto Laryngol..

[29] Mu Z.B., Huang Y.X., Zhao B.M., Liu Z.X., Zhang B.H., Wang Q.L. (2006). Effect of explosive noise on gastrointestinal transit and plasma levels of polypeptide hormones. World J. Gastroenterol..

[30] Alves-Pereira M., Ferreira J.M.R., de Melo J.J., Motylewski J., Branco N.A.A.C. (2003). Noise and the respiratory system. Rev. Port. Pneumol..

[31] Medina M.O., Barocio A.O., Montaño A.F., Ramos N.O. (2010). Discussing the effects of environmental noise upon the immune system. J. Acoust. Soc. Am..

[32] Ahmadi R., Gohari A., Hooshmand M. (2015). The effect of noise stress on serum levels of LH, FSH and testosterone in male rats. Feyz J. Kashan Univ. Med Sci..

[33] Aldo A.F., Luc P.J.S., Wolpert D.M. (2008). Noise in the nervous system. Nat. Rev. Neurosci..

[34] Helfrich R.F., Knight R.T. (2019). Cognitive neurophysiology: Event-related potentials. Handb. Clin. Neurol..

[35] Fabiani M., Karis D., Donchin E. (1990). Effects of mnemonic strategy manipulation in a Von Restorff paradigm. Electroencephalogr. Clin. Neurophysiol..

[36] Kutas M., Mccarthy G., Donchin E. (1977). Augmenting mental chronometry: The P300 as a measure of stimulus evaluation time. Science.

[37] Donchin M.C. (1981). A metric for thought: A comparison of P300 latency and reaction time. Science.

[38] Verleger R. (1997). On the utility of P3 latency as an index of mental chronometry. Psychophysiology.

[39] Reinvang I. (1999). Cognitive Event-Related Potentials in Neuropsychological Assessment. Neuropsychol. Rev..

[40] Mark K.M., Murphy D., Stevelink S.A.M., Fear N.T. (2019). Rates and Associated Factors of Secondary Mental Health Care Utilisation among Ex-Military Personnel in the United States: A Narrative Review. Healthcare.

[41] Babisch W. (2002). Stress hormones in the research on cardiovascular effects of noise. Noise Health.

